# HPV type-specific risks of high-grade CIN during 4 years of follow-up: A population-based prospective study

**DOI:** 10.1038/sj.bjc.6603843

**Published:** 2007-06-05

**Authors:** P Naucler, W Ryd, S Törnberg, A Strand, G Wadell, B G Hansson, E Rylander, J Dillner

**Affiliations:** 1 Department of Medical Microbiology, MAS University Hospital, Lund University, S-20502 Malmö, Sweden; 2 Department of Pathology and Clinical Cytology, Sahlgrenska University Hospital, 413 45 Göteborg, Sweden; 3 Cancer Screening Unit, Oncologic Centre, Karolinska Hospital, 171 76 Stockholm, Sweden; 4 Department of Medical Sciences, Dermatology and Venereology, University Hospital, 751 85 Uppsala, Sweden; 5 Department of Virology, University of Northern Sweden, 901 87 Umeå, Sweden; 6 Institute of Clinical Sciences, Department of Obstetrics and Gynecology, Karolinska Institute, Danderyd Hospital, 182 88 Stockholm, Sweden

**Keywords:** cervical cancer, screening, papillomavirus, attributable proportion, cohort studies

## Abstract

We followed a population-based cohort of 5696 women, 32–38 years of age, by registry linkage with cytology and pathology registries during a mean follow-up time of 4.1 years to assess the importance for CIN2+ development of type-specific HPV DNA positivity at baseline. HPV 16, 31 and 33 conveyed the highest risks and were responsible for 33.1, 18.3 and 7.7% of CIN2+ cases, respectively. Women infected with HPV 18, 35, 39, 45, 51, 52, 56, 58, 59 and 66 had significantly lower risks of CIN2+ than women infected with HPV 16. After adjustment for infection with other HPV types, HPV types 35, 45, 59 and 66 had no detectable association with CIN2+. In summary, the different HPV types found in cervical cancer show distinctly different CIN2+ risks, with high risks being restricted to HPV 16 and its close relatives HPV 31 and HPV 33.

Infection with ‘high-risk’ types of HPV is the major cause of cervical cancer, and the distribution of different HPV types in cancer tissue has been extensively analysed (Walboomers *et al*, 1999; Bosch *et al*, 2002; Munoz *et al*, 2003). Fifteen HPV types that infect the genital mucosa have been proposed as ‘high-risk’ HPV types as they have been found more often in cervical cancers than among healthy subjects (Munoz *et al*, 2003). Data on the HPV type-specific risk of cervical neoplasia has hitherto mostly been based on cross-sectional case–control studies (Bosch *et al*, 1995; Clifford *et al*, 2003; Munoz *et al*, 2003). Case–control studies are sensitive to several biases, notably selection bias, differential sampling bias and other reverse causality biases. Absolute risks and population attributable proportions for each HPV type form the basis for decisions regarding which HPV types should be included in HPV screening tests as well as in vaccines and it is therefore important to estimate the type-specific risks using population-based prospective studies, a study design that minimises major sources of bias.

Only two previous prospective studies have assessed the risk associated with several individual HPV types (Schiffman *et al*, 2005; Berkhof *et al*, 2006), while several prospective studies have assessed certain clusters of HPV types (Koutsky *et al*, 1992; Liaw *et al*, 1999; Sherman *et al*, 2003; Szoke *et al*, 2003; Peto *et al*, 2004; Winer *et al*, 2005) or only investigated HPV types 16 and 18 (Khan *et al*, 2005).

Therefore, we HPV-tested a population-based cohort of women and used a comprehensive registry-based follow-up to identify the risks for development of histopathologically verified high-grade cervical intraepithelial neoplasia (CIN2+) associated with infections with 14 different so-called ‘high-risk’ HPV types.

## MATERIALS AND METHODS

### Cohort definition

A population-based multicentre study was started in Sweden in May 1997 with the main purpose to evaluate the effect of HPV testing in primary cervical cancer screening. Women aged between 32 and 38 years (mean age: 35.1 years) in five regions in Sweden (Gothenburg, Malmö, Stockholm, Umeå and Uppsala) who took part in organised cervical screening were invited to take part in the study. Following informed consent, 12 527 women were enrolled and randomised either to action on HPV tests (6257 women) or to no action on HPV tests (6270 women), as described in detail elsewhere (Elfgren *et al*, 2005). All women had a cervical brush sample taken at baseline that was used for routine cytological screening and then frozen in 1 ml of 0.9% NaCl for future HPV DNA analysis. Referral to colposcopy was based on routine clinical management. Furthermore, HPV-positive women in the intervention arm that did not have an abnormal enrolment smear in cytology and pathology registries were invited for a second HPV test and cytology on average 19 months later and if persistently positive invited to colposcopy. A matched number of women from the control arm were also invited for HPV test, cytology and colposcopy (Elfgren *et al*, 2005). For the present study, a population-based cohort was formed from 6257 women in the intervention arm as well as 409 women randomly selected from the control arm that had HPV tests performed on their baseline samples.

All women were followed by registry linkages using unique personal identification numbers with both the regional cytology and pathology registries in the enrolling regions, as well as with the national cervical screening registry, to detect development of CIN2+. All women with an abnormal histopathological diagnosis as well as all women invited for colposcopy within the study protocol had their specimens re-evaluated by a single expert pathologist (WR) who was masked to the HPV status of the women. For 22 specimens that could not be located in the pathology archives, the original diagnosis was retained. Overall, 126/148 (85%) of the CIN2+ diagnoses in the present study have been confirmed by expert pathologist review. Women who had an inadequate (*β*-globin-negative) HPV test at baseline (172 women) or who had no cytological or histological samples registered during follow-up (796 women) were excluded from the analysis. The final population-based cohort thus consisted of 5696 women with a mean follow-up time of 4.1 years.

### HPV DNA testing

The virus laboratory that performed the HPV analyses was masked to cytological and histological diagnoses of the women. Cervical brush samples were analysed using a general HPV primer GP5+/6+-mediated PCR-enzyme immunoassay consisting of a pool of digoxigenin-labelled HPV type-specific oligonucleotide probes of 14 high-risk HPV types (types 16, 18, 31, 33, 35, 39, 45, 51, 52, 56, 58, 59, 66 and 68) (de Roda Husman *et al*, 1995; Jacobs *et al*, 1997). Human *β*-globin was amplified simultaneously in the PCR-EIA assay to test for sample DNA quality. All HPV-positive samples were typed by reverse dot blot hybridisation (RDBH) using HPV type-specific plasmids corresponding to the different HPV types tested for in the PCR-EIA (Forslund *et al*, 2002). PCR-EIA-positive samples negative in RDBH were cloned and sequenced. Samples were considered HPV positive only if successfully typed by RDBH or by DNA sequencing.

### Statistical analysis

Statistical analyses were performed using STATA 9.0. Absolute cumulative risks of future CIN2+ and CIN3+ with binominal exact 95% confidence intervals (CI) were calculated for each HPV type. Relative rates for CIN2+ and their 95% CI were calculated using Poisson regression. Women were censored at their last testing date, except for women who developed CIN2+ who were censored at the date when the diagnostic biopsy was taken. Since lesions that were detected early during follow-up might have been present already at baseline we split the data according to length of follow-up of more or less than 6 months. A likelihood ratio test was then performed to assess if there was interaction between HPV infection and time of CIN2+ diagnosis. There was weak evidence for interaction (*P*-value 0.10) and thus we adjusted for time of diagnosis by introducing a term in the regression model representing follow-up of more or less than 6 months. Because the risk associated with one HPV type can be confounded by co-infection with other HPV types, we adjusted for infection with other HPV types by including type-specific HPV data as single variables in a multivariate regression model. Population attributable proportions were calculated as p_c_^*^(RR-1)/RR where p_c_ is the proportion exposed to an HPV type among cases and RR is the type-specific relative rate adjusted for infection with other HPV types and censoring before or after 6 months of follow-up (Miettinen, 1974). Ninety-five percent CI for population attributable proportions were calculated from the standard error of the log-transformed complement of the population attributable proportions [ln(1-PAF)] (Rothman and Greenland, 1998). To test for type-specific differences of relative rates, the *β*-coefficients of each HPV type in the multivariate regression model were compared with the *β*-coefficient of HPV 16 by fitting a constrained model and performing a likelihood ratio test (StataCorp, 2005).

## RESULTS

During a mean follow-up time of 4.1 years, 148 women developed CIN2+. One hundred and twenty-seven (85.8%) of these had a positive HPV test at baseline. The HPV type with highest population-based prevalence of infection was HPV 16 (2.5%), followed by type 31 (1.4%), 45 (0.9%) and 18 (0.7%). A single HPV type was detected in 401 (87.0%) of the HPV-positive women, while 60 (13.0%) women had more than one virus type detected in the same sample. Most multiple HPV infections were infections with two HPV types, but up to four different HPV types could be detected in the same sample. The absolute risk of CIN2+ among HPV-positive women ranged from 0% for HPV 59 to 48.0% (95% CI: 27.8–68.7) for HPV 33 (Table 1). HPV 16 infection had an absolute risk of future CIN2+ of 36.6% (95% CI: 28.7–45.1) and HPV 18 infection had an absolute risk of 25.6% (95% CI: 13.0–42.1). HPV 31 and 58 were also associated with very high absolute risks of 36.7% (95% CI: 26.1–48.3) and 30.4% (95% CI: 13.2–52.9), respectively (Table 1). The absolute risk of developing CIN2+ among women infected with any high-risk HPV type was 27.5% (95% CI: 23.5–31.9). Women who were HPV negative had a low absolute risk of future CIN2+ (0.40% (95% CI: 0.25–0.61)). The absolute risks for future CIN3+ were highest for HPV 16 (28.2% (95% CI: 20.9–36.3)), HPV 33 (28.0% (95% CI: 12.1–49.4)), HPV 31 (22.8% (95% CI: 14.1–33.6)) and HPV 58 (21.7% (95% CI: 7.5–43.7)) (Table 1).

The relative rates of CIN2+ for each HPV type were assessed using women negative for the corresponding HPV type as the reference group (Table 1). It should be noted that we have deliberately avoided analyses using women who were HPV negative for all HPV types as the reference group. Such analysis cannot take the issue of multiple infections into account, resulting in inflated relative rates, for example the relative rate for HPV 16 was 27.6 (19.7–38.6) (Table 1), but the HPV 16-related relative rate would have been 118.4 (71.3–196.6) if women negative for all HPV types had been used as a reference group.

After adjustment for concomitant infections with other HPV types, the type-specific relative rates segregated into three groups. HPV 16, 31 and 33 had very high relative rates (exceeding 10) (Table 1), with all other types having significantly lower risks than HPV 16 (Table 1). HPV types 18, 39, 51, 52, 56 and 58 conferred significantly elevated risks for CIN2+, with relative risks in the range 3- to 7-fold (Table 1). We were not able to detect any excess risk for CIN2+ associated with HPV 35, 45, 59 or 66 (Table 1).

Population attributable proportions were based on the type-specific relative rates adjusted for all other HPV types. HPV 16 attributed to 33.1% (95% CI: 24.7–40.6) followed by HPV 31 (18.3% (95% CI: 11.5–24.5)), HPV 33 (7.7% (95% CI: 3.1–12–0)) and HPV 18 (5.7% (95% CI: 1.5–9–7)) (Table 1). HPV 35, 39, 45, 56, 59 and 66 each contributed to 2.0% or less of CIN2+ in the population. HPV 16 and 18 together attributed to 39.0% (95% CI: 30.2–46.7) of CIN2+ and the four types (HPV 16, 18, 31, 33) that contributed most individually jointly attributed to 64.0% (95% CI: 55.0–71.2). Overall, 84.7% (95% CI: 73.3–89.7) of CIN2+ was attributed to an HPV infection.

## DISCUSSION

This prospective large-scale study provides individual estimates of the risk for future histopathologically confirmed high-grade CIN for each one of 14 genital HPV types, commonly included in ‘high-risk’ HPV tests. The large size and prospective study design, nested within the national screening programme and our comprehensive follow-up argue in favour of reliability of estimates.

We report that several HPV types previously classified as ‘high-risk’ types (Munoz *et al*, 2003) convey significantly lower risk for future CIN2+ compared to HPV 16. This is in line with a cohort study in Costa Rica of 10 000 women which reported HPV 16 to be uniquely carcinogenic among ‘high-risk’ HPV types in its risk of progression to CIN3/cancer after persistent infection (Schiffman *et al*, 2005). Also, in a cohort of 20 810 women in Portland, US, the 10-year cumulative incidence rate of CIN3+ among women with a negative, equivocal, or mildly abnormal baseline cervical pap smear was 17.2% among HPV 16-positive women, 13.6% among HPV 18-positive women but only 3.0% among women with a positive Hybrid Capture 2 test but negative for HPV 16 and 18 at baseline (Khan *et al*, 2005). Besides HPV 16 we also found that HPV 31 and 33 conveyed rate ratios of future CIN2+ above 10. These results are similar to a Dutch report, where these three types were associated with the highest risk for future CIN2+ during 18 months of follow-up (Berkhof *et al*, 2006).

Risk classification of genital HPV types has hitherto mostly been based on case–control studies (Munoz *et al*, 2003). In a pooled analysis of 11 case–control studies performed by IARC that included samples from 1918 women with squamous cervical carcinoma and 1928 controls, 15 HPV types (HPV 16, 18, 31, 33, 35, 39, 45, 51, 52, 56, 58, 59, 68, 73 and 82) were classified as high-risk types and HPV 26, 53 and 66 as probably high-risk types (Munoz *et al*, 2003). Even though prospective studies use high-grade CIN as end point, which is only a surrogate for cervical cancer, they convey important methodological advantages to establish causal relationships. Case–control studies are not only sensitive to reverse causality bias, but many case–control studies sample differently from cases (biopsies) and controls (brush samples) which might affect HPV test performance. Furthermore, it is often difficult to obtain population-representative controls. Therefore we believe that more robust risk classification of HPV types can be obtained from prospective population-based studies.

Population-attributable proportions estimate the reduction in incidence of a disease if the exposure under study was eliminated from the population (e.g., by vaccination). The population-attributable proportions were based on HPV type-specific relative rates, that is the rates for a specific HPV type compared to the rate among women negative for that HPV type (not compared to women negative for all HPV types). This choice of reference group is more appropriate for studying attributable proportions since the elimination of one HPV type does not imply that the population would become negative for all HPV types. It should be noted that even though our type-specific relative rates as well as our classification of which types are oncogenic differs from the above-mentioned studies (Munoz *et al*, 2003), our relative rates, if we would have used women negative for any HPV as reference group would have been rather similar to these other studies (data not shown).

After adjustment of our type-specific estimates for presence of other HPV types, we found no elevated risk for future CIN2+ among women infected with HPV 35, 45, 59 and 66. However, the CI were large, especially for HPV 35, and it is possible that our failure to detect any association with disease for these HPV types may be attributable to limited statistical power. We found that the elimination of HPV 16 would diminish the burden of CIN2+ by 33% and removal of both HPV 16 and 18 would reduce the incidence of CIN2+ by 39% in our population of Swedish women aged 32–38 years. It is noteworthy that both HPV 31 and 33, which are not included in current prophylactic vaccines, both contributed to more CIN2+ than did HPV 18. These four HPV types (HPV 16, 18, 31 and 33) jointly attributed to almost 65% of all CIN2+, and prevention of infection with these four types would therefore be most important. By contrast, HPV types 35, 39, 45, 56, 59 and 66 each contributed to 2.0% or less of CIN2+ in the population and inclusion of these types in HPV vaccines therefore seems less important, as the theoretical possibility exists that inclusion of too many HPV types in second generation HPV vaccines might impair the response against the most important HPV type (i.e., HPV 16). In a cohort of 10 000 women in Costa Rica, HPV 16 was the type that contributed most to prevalent cases of CIN3+ followed by HPV 58, 18 and 31 (Schiffman *et al*, 2005). In our study, HPV 58 infection was rare in the population and only 4% of CIN2+ cases were attributed to HPV 58. Since population attributable proportions are dependent not only on the type-specific relative risks but also on the type-specific prevalence, it is likely that differences in type-specific prevalences of infection in different populations account for the observed differences.

Limitations of the study include the risk of verification bias, as women with a negative HPV test are less likely to be referred to colposcopy and subsequently having a biopsy taken during the course of the study. This could influence the attributable proportion estimates but not the relative comparison of type-specific absolute risks or risk rates. The bias of HPV-based verification diminishes successively with increasing length of follow-up using conventional cytology and most of the women in the cohort were followed for more than two screening rounds (in Sweden the screening interval is 3 years). Also, detection of CIN2+ is exceedingly rare when HPV-negative, cytology-negative women are referred to colposcopy suggesting that verification bias has not materially affected our estimates (Belinson *et al*, 2001).

It has been suggested that CIN 3+ is a more appropriate proxy for the risk of future cervical cancer than CIN2+ since the regression rate is higher for CIN2 than for CIN 3 (Schiffman and Kjaer, 2003). Although our statistical power to address CIN3+ rates was more limited, we found similar patterns of differences in HPV-type-specific risks.

In conclusion, we found that different so-called ‘high-risk’ HPV types convey very different risks for future CIN2+, with HPV 16 and its close relatives HPV 31 and 33 consistently conferring the highest risks. Appreciation of the HPV-type-specific risks for CIN2+ can be helpful for interpretation and design of HPV tests as well as for second-generation vaccines against HPV.

## Figures and Tables

**Table 1:** HPV type-specific risks and population attributable proportions for the development of histopathologically confirmed high-grade CIN during a mean follow-up time of 4 years in a cohort of 5696 women

**HPV type**	**Absolute risk of CIN2+ ((95% C), CIN2+ cases/women infected)**	**Absolute risk of CIN3+ ((95% CI), CIN3+ cases/women infected)**	**Relative rate^a^ for CIN 2+ (95% CI)**	**Relative rate^b^^,^^c^ for CIN 2+ (95% CI)**	**Population attributable proportion (95% CI)**
HPV negative	0.40% (0.25–0.61) 21/5235	0.19% (0.092–0.35) 10/5235	—	—	—
HPV 16	36.6% (28.7–45.1) 52/142	28.2% (20.9–36.3) 40/142	27.6 (19.7–38.6)	17.4 (12.0–25.1)	33.1% (24.7–40.6)
HPV 18	25.6% (13.0–42.1) 10/39	15.4% (5.9–30.5) 6/39	11.3 (6.0–21.5)	6.3 (3.1–12.5)^*^	5.7% (1.5–9–7)
HPV 31	36.7% (26.1–48.3) 29/79	22.8% (14.1–33.6) 18/79	20.1 (13.4–30.1)	14.9 (9.7–22.9)	18.3% (11.5–24.5)
HPV 33	48.0% (27.8–68.7) 12/25	28.0% (12.1–49.4) 7/25	27.8 (15.4–50.2)	18.5 (9.1–37.4)	7.7% (3.1–12–0)
HPV 35	9.1% (1.1–29.2) 2/22	4.5% (0.1–22.8) 1/22	4.2 (1.0–16.9)	3.1 (0.8–12.9)^**^	0.9% (0–2.8)
HPV 39	17.6% (3.8–43.4) 3/17	11.8% (1.5–36.4) 2/17	6.6 (2.1–20.7)	6.0 (1.9–19.4)^*^	1.7% (0–3.9)
HPV 45	19.2% (9.6–32.5) 10/52	7.7% (2.1–18.5) 4/52	8.3 (4.4–15.8)	0.9 (0.4–2.1)^***^	—
HPV 51	17.2% (5.8–35.8) 5/29	17.2% (5.8–35.7) 5/29	7.4 (3.0–18.0)	5.3 (1.9–14.7)^**^	2.7% (0–5.6)
HPV 52	26.1% (10.2–48.4) 6/23	13.0% (2.8–33.6) 3/23	10.9 (4.8–24.6)	4.2 (1.8–9.7)^**^	3.1% (0–6.3)
HPV 56	10.3% (2.9–24.2) 4/39	2.6% (0.06–13.5) 1/39	4.3 (1.6–11.7)	3.7 (1.3–10.2)^**^	2.0% (0–4.6)
HPV 58	30.4 (13.2–52.9) 7/23	21.7% (7.5–43.7) 5/23	13.4 (6.3–28.7)	5.9 (2.5–14.1)^*^	3.9% (0.4–7.3)
HPV 59	0% 0/14	0%	0	0^***^	—
HPV 66	7.4% (0.9–24.3) 2/27	3.7% (0.0 –19.0) 1/27	3.1 (0.8–12.4)	0.3 (0.07–1.3)^***^	—
HPV 68	No detected infection	—	—	—	—
HPV 16 and/or HPV 18	34.3% (27.3–41.7) (61/178)	25.3% (19.1–32.3) 45/178	27.4 (19.7–38.0)	18.9 (13.2–27.1)	39.0% (30.2–46.7)
HPV16 and/or 18 and/or 31 and/or 33	56.1% (48.3–63.6) 97/270	24.4% (19.4–30.0) 66/270	47.2 (33.6–66.2)	42.4 (29.9–60.3)	64.0% (55.0–71.2)
All HPV positives	27.5% (23.5–31.9) 127/461	18.4% (15.0–22.3) 85/461	80.8 (50.9–128.3)	80.8 (50.9–128.3)	84.7% (73.3–89.7)

a Adjusted for censoring before or after 6 months of follow-up.

b Adjusted for censoring before or after 6 months of follow-up and for other HPV types.

c Type-specific relative rates that were significantly different from HPV 16 relative rate: *P*-value ^*^0.01<0.05, ^**^0.001<0.01 and ^***^<0.001.
